# Cervical Epidural Anesthesia in the Management of a Patient With Breast Cancer With Cardiac Dysfunction: A Case Report

**DOI:** 10.7759/cureus.60074

**Published:** 2024-05-11

**Authors:** Amreesh Paul, Amol Singam, Nikhil Bhalerao, Dnyanshree Wanjari, Anjali Borkar

**Affiliations:** 1 Anesthesiology, Jawaharlal Nehru Medical College, Datta Meghe Institute of Higher Education & Research, Wardha, IND

**Keywords:** cancer pain, cardiac dysfunction, modified radical mastectomy (mrm), breast cancer, cervical epidural anesthesia

## Abstract

As one of the most common cancers in the world, breast cancer management is fraught with difficulties. Modified radical mastectomy (MRM) is one of the surgical procedures that is essential to the treatment of breast cancer. Cardiovascular issues, especially a reduced ejection fraction (EF), make these procedures more complex. Due to their increased vulnerability to adverse cardiac events during surgery, it is imperative to preserve hemodynamic stability and reduce physiological stress responses in these patients. A promising option in this changing field of anesthetic techniques is cervical epidural anesthesia (CEA). It effectively reduces hemodynamic fluctuations frequently linked to general anesthesia while providing analgesia. We report the case of an elderly patient with decreased EF and breast cancer scheduled for an MRM. To ensure the best possible outcomes in complex cases, the case report covers preoperative assessment, anesthesia technique, intraoperative management, and postoperative outcomes. This highlights the critical significance of customizing anesthesia and surgical procedures, informed consent, and meticulous postoperative pain management, and ultimately advocates for the broader implementation of CEA in such settings.

## Introduction

Breast cancer is a widespread malignancy globally that poses a complex set of challenges in management [1]. One of the mainstays of comprehensive treatment for breast cancer typically involves surgical treatments like modified radical mastectomy (MRM) [2]. Even though these surgeries are vital in managing the disease, they can pose a very challenging situation for the medical professionals involved in the treatment process when these individuals have concurrent cardiovascular conditions, especially if they have a poor ejection fraction (EF). Individuals with poor EF and ischemic heart disease are more likely to experience detrimental cardiac events during surgery, which can include potentially fatal cardiac arrest. For these patients, maintaining forward cardiac output by maintaining normovolemia, lowering afterload, and refraining from myocardial depressant medication use are the main anesthetic goals [3]. A particularly delicate time exists during the perioperative period of such patients when the physiological stress reaction to pain and surgery might worsen underlying heart problems. It is crucial to preserve hemodynamic stability throughout these surgical procedures to reduce the possibility of postoperative complications and improve patient outcomes [4]. Cervical epidural anesthesia (CEA) has become an intriguing technique in the changing landscape of anesthetic procedures, particularly in addressing the unique issues presented by patients with cardiac comorbidities [5]. This method of administering regional anesthesia can provide highly efficient analgesia while reducing the hemodynamic changes frequently linked to general anesthesia. Its capacity to enhance patient safety and comfort during complicated oncologic procedures like the MRM explains its rising popularity [6].

In this regard, we report the case of a breast cancer patient with low EF posted for an MRM. Our objective was to demonstrate that CEA can be used effectively in place of traditional general anesthesia, shedding light on potential benefits for individuals with compromised cardiac function. The whole spectrum of medical treatment is reviewed in this case study, including the preoperative evaluation, the anesthetic technique, intraoperative care, and the postoperative outcomes. In managing patients with cardiac comorbidities, we emphasize the significance of meticulous assessment of several variables and customized anesthetic preparation. By doing this, we hope to increase patient comfort and safety during surgical procedures. We also hope to provide insights to improve the changing perioperative care landscape for patients with reduced cardiac function.

## Case presentation

**Figure 1:**
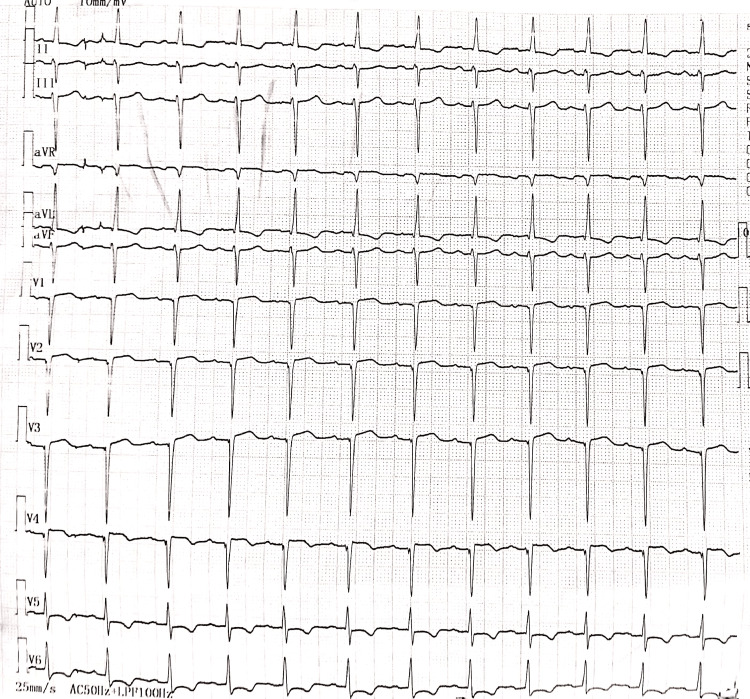
Electrocardiograph of the patient Electrocardiography revealed left axis deviation, left ventricular hypertrophy with a strain pattern, and ST segment depression in the lateral leads.

An 80-year-old female came with complaints of a lump on the side of her left breast for three years, associated with serous discharge from the lump for six months. The lump was 5 × 5 cm in size, hard in consistency, with associated erythema, and not adherent to the pectoralis muscle. The left axillary lymph nodes were palpable. The development of the lump was insidious in onset, gradual in progression, and associated with pain. She also gave a history of a weight loss of seven kilograms in the last year. She gave no history of fever, breathlessness, abdominal pain, or any bowel or bladder complaints. Fine needle aspiration cytology of the lump revealed invasive ductal carcinoma of the breast. The patient was scheduled for an MRM and, hence, sent to the preanesthetic suite of our hospital for assessment. A detailed medical history of the patient revealed a history of hypertension for seven years and a history of tobacco chewing for 50 years. She gave a history of taking telmisartan 40 mg irregularly. The patient also revealed having dyspnea on mild exertion - New York Heart Association (NYHA) class III. At the time of the assessment, the pulse rate was 82/min, blood pressure was 100/60 mmHg, respiratory rate was 17/min, and oxygen saturation was 93% in room air. Systemic examination revealed regular S1 and S2 sounds and normal vesicular breath sounds on auscultation. She gave no history of previous cardiac events, previous surgeries, or anesthetic exposures. Routine blood investigations revealed a hemoglobin of 9.8 g/dl, normal leukocyte and platelet counts, normal renal and hepatic function tests, and normal coagulation studies. Chest X-ray showed cardiomegaly, and electrocardiography revealed left axis deviation, left ventricular hypertrophy with strain pattern, and ST segment depression in the lateral leads, as seen in Figure 1.

**Figure 2:**
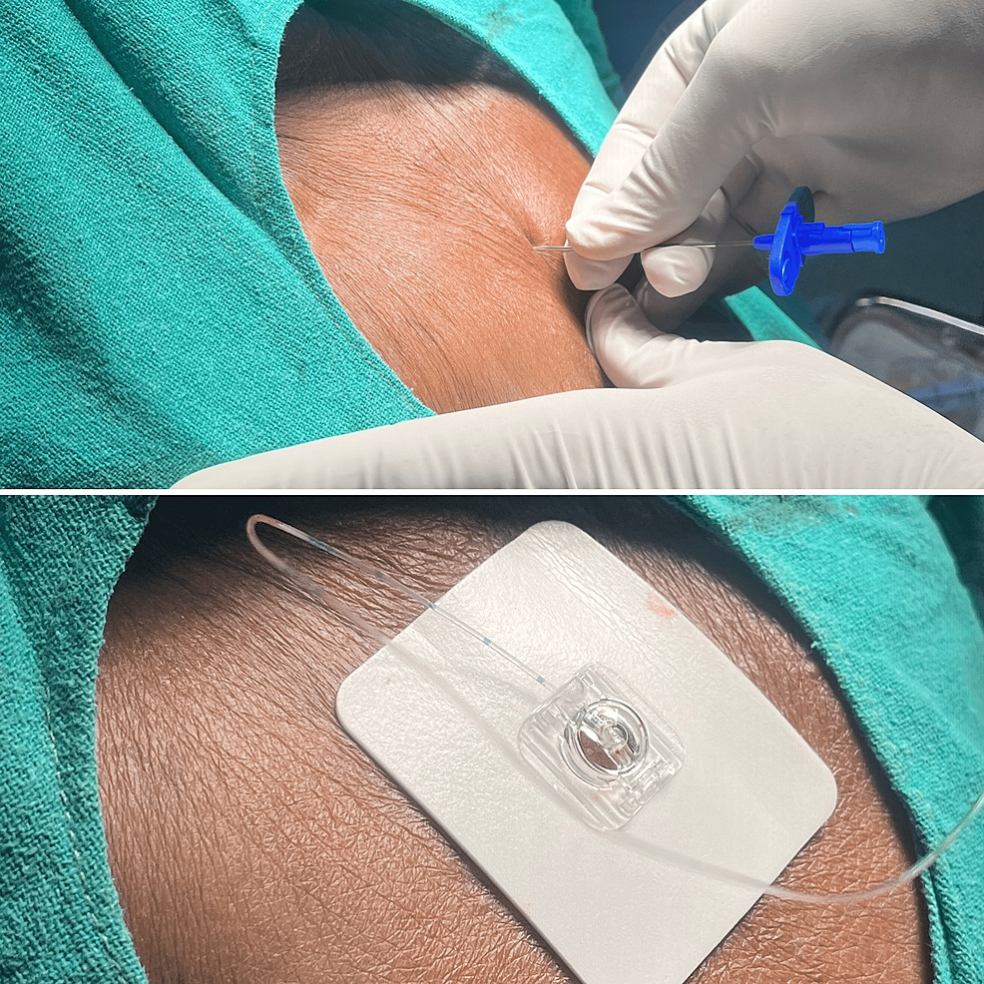
Administration of CEA in situ CEA, cervical epidural anesthesia

Hence, it was advised to obtain an echocardiography, which revealed a dilated left ventricle, mild global hypokinesia, and moderate left ventricular systolic dysfunction (EF: 43%). The patient was started on inj. heparin 5000 units thrice a day for these findings, which was stopped six hours before the scheduled time of the surgery. After a discussion with the onco-surgery team, it was decided to use CEA for the conduct of the surgery, with a catheter placement for postoperative analgesia. The patient and their relatives have explained the anesthetic technique in detail, including risks and benefits, and informed written consent was obtained, with general anesthesia being the backup plan. The patient was kept nil per os for six hours for solids and two hours for liquids before the proposed surgery. On the day of the surgery, the patient was shifted to the preoperative suite, baseline vital parameters were recorded, and an 18-gauge IV cannula was inserted in the right forearm. The patient was shifted into the operating suite, and multiparameter monitors were attached as per the American Society of Anesthesiologist’s standards. The pulse rate was 82/min, blood pressure was 120/80 mmHg, and oxygen saturation was 96% on room air. IV administration of Ringer’s lactate was started for the patient, and injections of IV ondansetron (4 mg), IV midazolam (2 mg), and IV ceftriaxone (1 g) were given. With the patient in the sitting position, under strict aseptic precautions, CEA was administered at the C7-T1 interspace with the head flexed on the thorax, as seen in Figure 2. The identification of the epidural space was done using the hanging drop technique with the use of 1 ml of normal saline. Once the saline was sucked in due to negative pressure, the epidural catheter was inserted, 3 ml of lignocaine-adrenaline (1:2,00,000) was administered, and the patient was positioned supine. No hemodynamic variations were noted for five minutes, after which the patient was gradually administered 8 ml of 0.25% bupivacaine. Post-CEA, blood pressure was 100/60 mmHg, and hence 500 mL of Ringer’s lactate was given, after which the blood pressure increased to 120/70 mmHg. Fluid maintenance with Ringer’s lactate was given throughout the surgery. The level of sensory blockade was assessed using the pinprick method and was found to be C3-T8, and the surgery was commenced.

**Figure 3:**
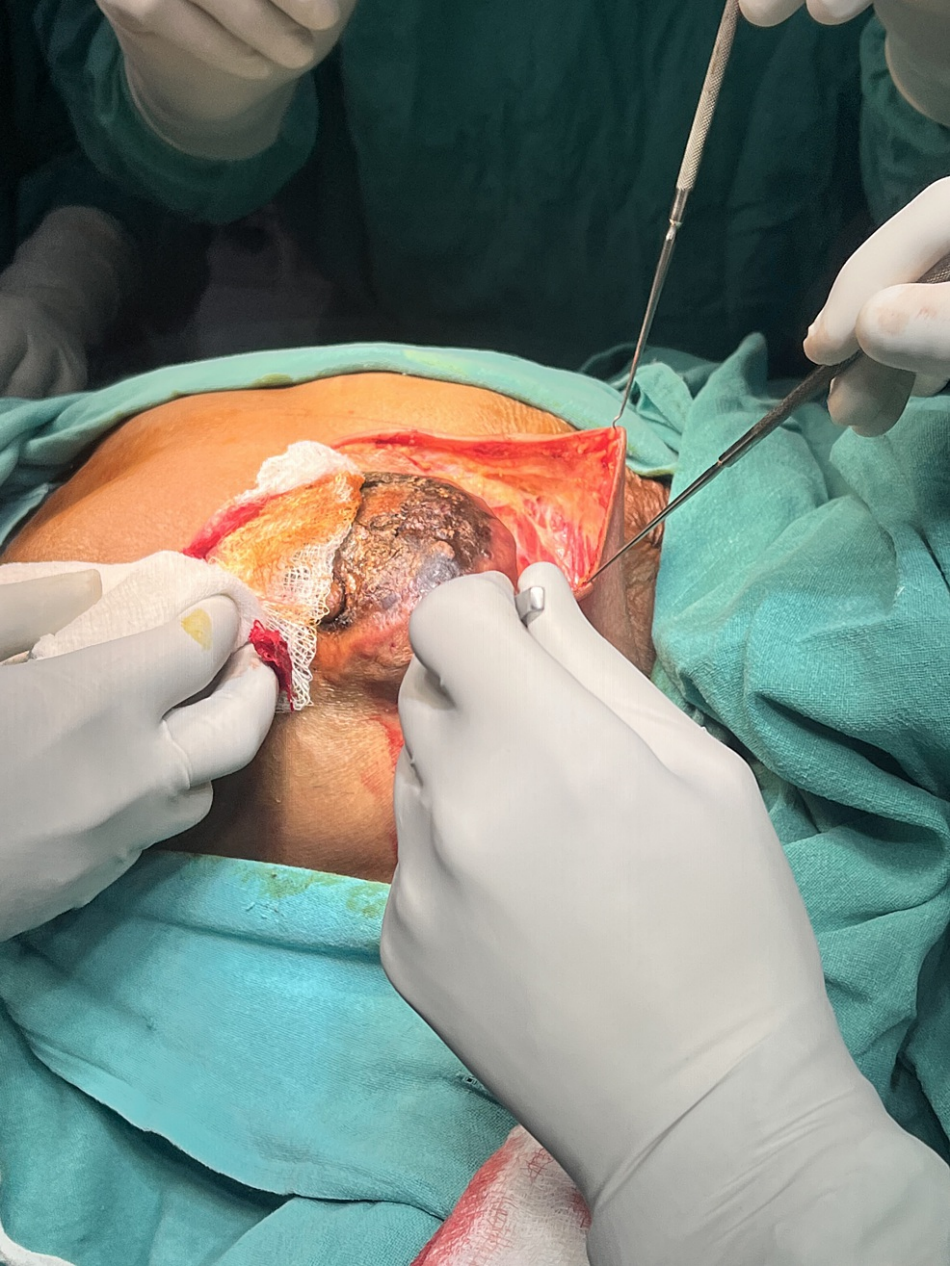
Intraoperative image Stewart’s incision, a transverse elliptical incision, was used for the surgery.

While keeping the ipsilateral arm abducted at a right angle, Stewart’s incision, a transverse elliptical incision, was made, encircling the nipple and the skin surrounding the tumor, as seen in Figure 3. The incision was deepened, and by using electrocautery, the upper flap was elevated in the subcutaneous plane.

**Figure 4:**
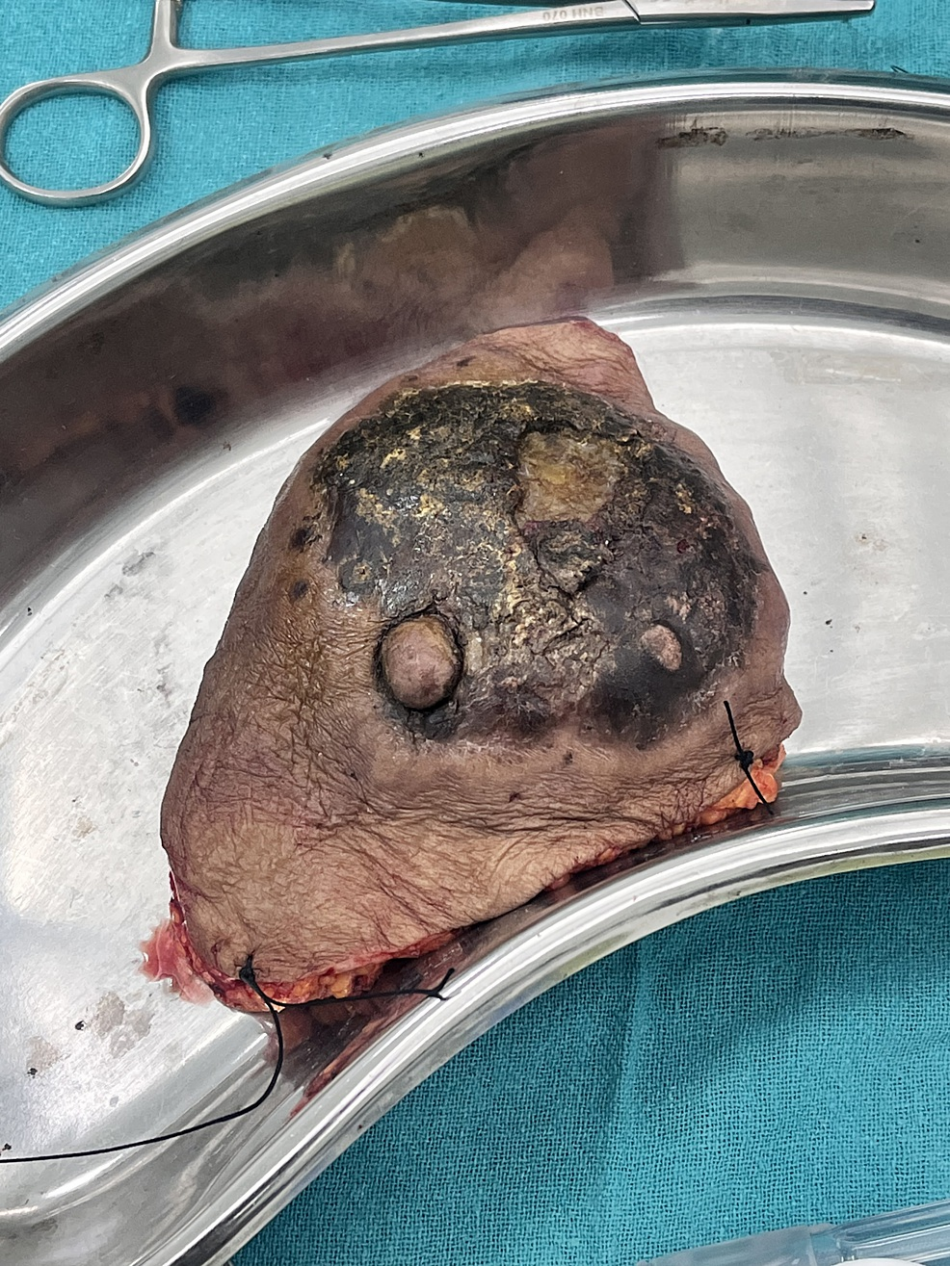
Excised breast tissue Breast tissue, including the pectoralis fascia, was meticulously separated from the pectoralis muscle and removed.

**Figure 5:**
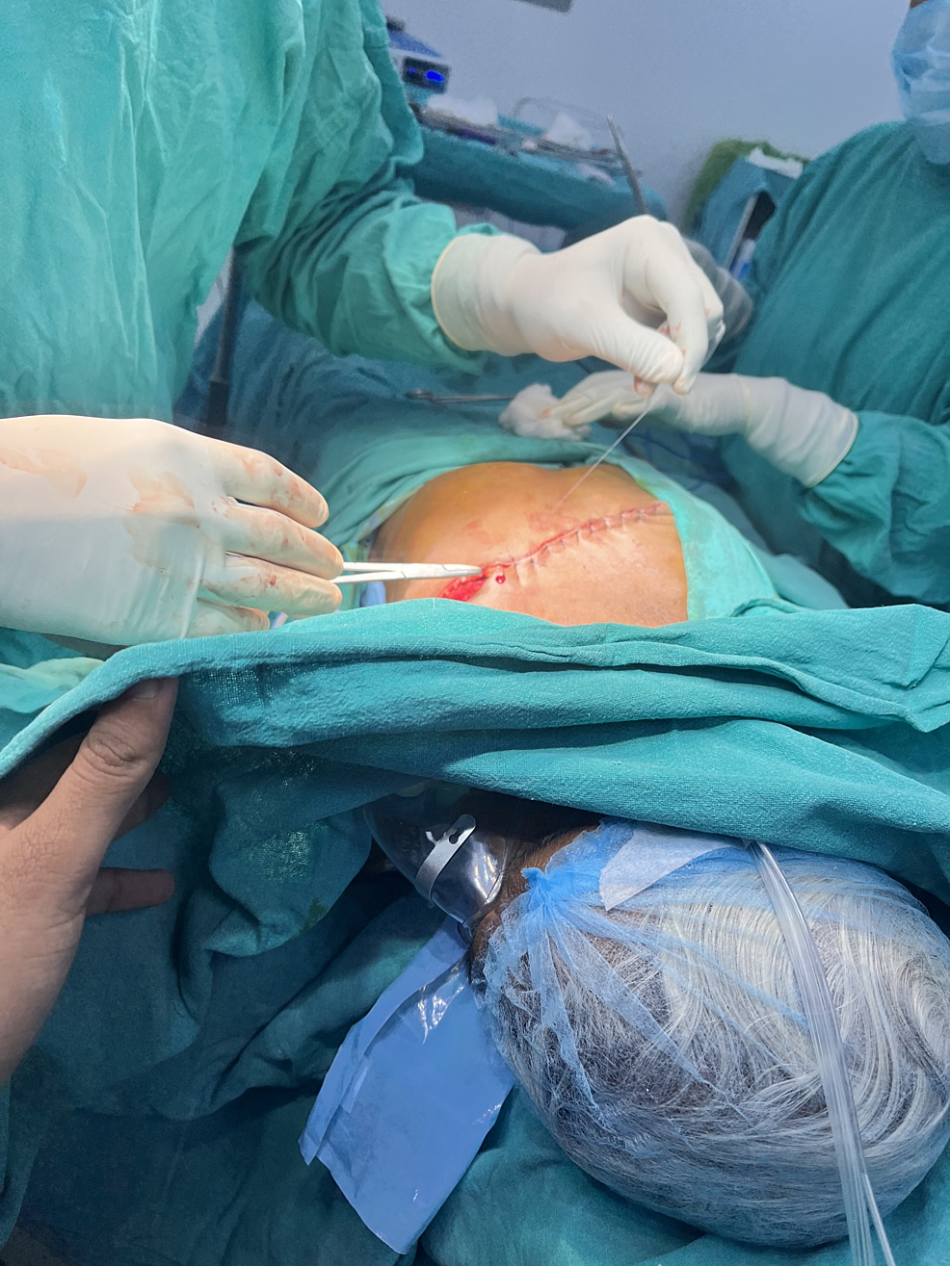
Image taken during wound closure showing the patient’s spontaneous breathing

The dissection extended laterally toward the lateral border of the latissimus dorsi muscle, superiorly to the clavicle, and medially to the sternum. Breast tissue, including the pectoralis fascia, was meticulously separated from the pectoralis muscle. The lateral border of the pectoralis minor muscle was visible once the pectoralis major muscle was retracted. An incision was made parallel to the axillary vein in the clavipectoral fascia that covers it. The entirety of axillary fat at levels I and II, along with its contents, was separated from the vein below. The long thoracic nerve of Bell, which supplies the serratus anterior muscle, the latissimus dorsi pedicle, and its accompanying thoracodorsal nerve were identified and preserved. Similarly, the lower flap was elevated in the subcutaneous plane until the inferior rectus sheath was reached. The level I and II axillary nodes and the whole breast were removed, as seen in Figure 4. The adipose tissue surrounding the interpectoral lymph node was removed. Hemostasis was verified to be adequate. To regulate postoperative drainage, a drain was installed. The surgical site was meticulously closed in layers to guarantee appropriate wound closure, as seen in Figure 5. The surgery was concluded in 75 minutes, and no additional boluses of epidural drug, analgesia, or intraoperative sedation were required.

The patient was then shifted to the postanesthesia care unit, monitored for six hours, and then shifted to the ward. Pain scores were assessed using the Visual Analogue Scale, and they were found to be 0, 0, 1, 2, 2, 3, 4, 2, and 3 at first, second, fourth, eighth, 12th, 16th, 20th, and 24th-hour post-epidural anesthesia administration. Moreover, 8 ml of 0.125% bupivacaine was administered through the epidural catheter around the 20th postoperative hour, with close monitoring of blood pressure and pulse rate. The patient was comfortable in the postoperative period and did not require additional top-ups of epidural analgesia or IV analgesics. On postoperative day 1, she was started back on heparin, which was stopped six hours prior to removing the epidural catheter on postoperative day 2. The patient was started on tablets of clopidogrel 75 mg, aspirin 75 mg, and atorvastatin 20 mg (once a day) as per the advice of the cardiologist, and was discharged from the ward on postoperative day 3.

## Discussion

In the field of oncology, the management of breast cancer - more particularly, the approach to anesthesia and analgesia - is a subject of growing significance and interest. General anesthesia has historically been used for radical mastectomies. However, new advancements in the discipline have caused this method to be reconsidered [7]. With several potential benefits, CEA is becoming a competitive alternative to breast procedures, including mastectomy [8]. Economical factors can lead to more cost-effective healthcare, which is one noteworthy benefit. CEA is a desirable alternative for patients and healthcare professionals since it also provides good postoperative analgesia and has been linked to decreased postoperative morbidity [9].

An elderly woman with underlying cardiac dysfunction and breast cancer is the subject of this case study, which demonstrates the comprehensive care of a challenging clinical condition. A multidisciplinary team needs to collaborate to maximize patient care and safety, as shown by the diverse strategy used in this instance. The patient’s poor EF (43%) on echocardiography, indicative of underlying heart failure, presented a significant problem for general anesthesia. Intraoperative cardiac events are more common in patients with impaired heart function, and more care needs to be undertaken to reduce these risks. It was concluded that a safer alternative to general anesthesia would be CEA. CEA has many advantages when administered to patients with heart failure. It provides adequate analgesia while minimizing the hemodynamic alterations often associated with general anesthesia. Additionally, this technique minimizes the requirement for IV analgesics, which may be detrimental to the cardiovascular system [5,8]. In these situations, informed consent is crucial, just like in any complex case. The anesthetic method, its side effects, and its benefits were fully explained to the patient and their relatives. By taking this action, the patient was allowed to participate in their care while ensuring transparency. The patient’s postoperative comfort and recovery, including the assessment of pain levels, demonstrate the success of this technique. Prevention of complications and early mobilization is dependent on effective postoperative pain management.

In contrast to previous concerns about CEA challenges, new studies indicate that it is a safe and valuable substitute, especially for neck, breast, and upper limb procedures [8,10-12]. The successful application of CEA in situations such as the one described in this study, where it produced ideal surgical conditions, has been made possible by this shift in attitude. With CEA, the cervical epidural space is injected with local anesthetics to block the cervical nerve roots. Since its introduction in 1933, this technique has undergone refinement and evolution for upper thoracic surgery. Because the brachial plexus (C5-C8) innervates the pectoralis muscle, CEA for breast cancer surgery is frequently administered in the C6-T1 epidural region [13]. When local anesthetics are utilized, CEA provides a wider sensory blockade from C3 to T8, which is helpful for axillary dissection. CEA can help improve perioperative results and reduce morbidity by enabling specific procedures without general anesthesia, particularly in high-risk patients [14].

## Conclusions

A significant development in the field is the reevaluation of anesthesia methods for breast cancer surgery, particularly the introduction of CEA. This modification is in line with the objectives of improving pain management for patients having breast surgery, decreasing postoperative morbidity, and improving patient care. Given the high prevalence of breast cancer, implementing cutting-edge methods like CEA can significantly improve patient outcomes and the state of healthcare. The significance of a multidisciplinary approach is emphasized in this case study while handling complicated instances involving elderly people with cardiac issues. To ensure the best possible outcome for patients having surgical procedures for breast cancer, it emphasizes the importance of meticulous assessment, tailored anesthesia and surgical techniques, and careful postoperative pain management. The favorable result in this instance shows the possible advantages of CEA in such a setting.

## References

[1] Sun YS, Zhao Z, Yang ZN (2017). Risk factors and preventions of breast cancer. Int J Biol Sci.

[2] Wright E, Lee GK, Torre DL (2018). Breast reconstruction. The Breast: Comprehensive Management of Benign and Malignant Diseases.

[3] Chua JH, Nguyen R (2015). Anesthetic management of the patient with low ejection fraction. Am J Ther.

[4] Hedge J, Balajibabu PR, Sivaraman T (2017). The patient with ischaemic heart disease undergoing non cardiac surgery. Indian J Anaesth.

[5] Sanchez JI (2016). Cervical epidural blockade: a technique in disuse or alternative to other techniques. J Anesth Crit Care.

[6] Kulkarni K, Namazi IJ, Deshpande S, Goel R (2013). Cervical epidural anaesthesia with ropivacaine for modified radical mastectomy. Kathmandu Univ Med J (KUMJ).

[7] Sherwin A, Buggy DJ (2018). Anaesthesia for breast surgery. BJA Educ.

[8] Singh AP, Tewari M, Singh DK, Shukla HS (2006). Cervical epidural anesthesia: a safe alternative to general anesthesia for patients undergoing cancer breast surgery. World J Surg.

[9] Li J (2014). Regional anesthesia for acute pain management in elderly patients. World J Anesthesiol.

[10] Altaiey AM (2022). Cervical epidural anesthesia for large goiter with tracheal deviation. J Res Med Dent Sci.

[11] Elisabet Nystrom UM, Nystrom NA (1997). Continuous cervical epidural anesthesia in reconstructive hand surgery. J Hand Surg.

[12] Menon M, Taha N, Purohit N, Kothari V, Singh S (2016). Continuous cervical epidural analgesia in metastatic spinal cord compression. Indian J Palliat Care.

[13] Patel A (2021). Cervical epidural anesthesia as a sole technique in breast cancer with multiple comorbidities. Anesth Clin Res.

[14] Wenk M, Massoth C, Pöpping DM, Möllmann M (2017). Feasibility of cervical epidural anesthesia for breast cancer surgery. Anesthesiol Res Pract.

